# Mitogen-Activated Protein Kinase Phosphatase-2 Deletion Promotes Hyperglycemia and Susceptibility to Streptozotocin-Induced Diabetes in Female Mice In Vivo

**DOI:** 10.3390/cells14040261

**Published:** 2025-02-12

**Authors:** Nabin Ghimire, Morgan Welch, Cassandra Secunda, Alexis Fink, Ahmed Lawan

**Affiliations:** Department of Biological Sciences, University of Alabama in Huntsville, Huntsville, AL 35899, USA; ng0063@uah.edu (N.G.); mmw0025@uah.edu (M.W.); cs0244@uah.edu (C.S.); af0092@uah.edu (A.F.)

**Keywords:** hyperglycemia, diabetes, MKP-2, MAPK, islet

## Abstract

The development of type 2 diabetes (T2D) is largely dependent on the maintenance of pancreatic islet function and mass. Sexual dimorphism in T2D is evident in many areas, such as pathophysiology, treatment, and prevention. Mitogen-activated protein kinase phosphatase-2 (MKP-2) has a distinct role in the regulation of cell proliferation and the development of metabolic disorders. However, whether there is a causal relationship between MKP-2 and diabetes onset is unclear. The aim of this study was to determine the role of MKP-2 in the regulation of whole-body glucose homeostasis and the impact on pancreatic islet function using streptozotocin-induced pancreatic injury. Here, we show that female mice with whole-body deletion of MKP-2 exhibit hyperglycemia in mouse models treated with multiple low doses of streptozotocin (STZ). In comparison, both male MKP-2 wild-type and knockout mice were hyperglycemic. Consistent with the hyperglycemia, female MKP-2-deficient mice exhibited reduced islet size. Under T2D conditions, MKP-2-deficient mice display enhanced pancreatic JNK and ERK phosphorylation that is associated with the downregulation of genes important for pancreatic islet development and function, Pdx-1 and MafA. Furthermore, we found impaired metabolic flux in adipose tissue that is consistent with hyperglycemia and dysfunctional pancreas. MKP-2 deletion results in reduced Akt activation that is associated with increased adiposity and insulin resistance in female MKP-2 KO mice. These studies demonstrate the critical role of MKP-2 in the development of T2D diabetes in vivo. This suggests that MKP-2 may have a gender-specific role in diabetes development. This discovery raises the possibility that postmenopausal prevention of T2D may benefit from the activation of MKP-2 activity in islet cells.

## 1. Introduction

Diabetes, the most prevalent metabolic disease, is currently the seventh leading cause of death in the US and globally [1,2]. Elevated plasma glucose levels cause diabetes, which can be attributed to either autoimmune destruction of pancreatic β-cell type 1 diabetes (T1D) or obesity and insulin resistance type 2 diabetes (T2D) [3,4]. Insulin secretion by pancreatic β-cells is crucial in both T1D and T2D. Apoptosis leads to insulin dependence in diabetes, while insulin replacement therapy reduces effectiveness due to the loss of functional islet mass from apoptosis [5]. Glucose homeostasis varies significantly by gender. In healthy subjects, women may be more insulin sensitive than men, have higher circulating insulin levels, and clear glucose more efficiently [6,7]. The reason is still unclear. According to reports on pancreatic biopsies, women have an approximately 6% higher number of β-cells than men, which is consistent with the increased insulin secretion following oral glucose tolerance test (OGTT) [8,9]. Although men are more likely than women to have diabetes worldwide, women are more likely than men to have the disease [2,10]. This opinion is supported by the majority of available data in many rodent models as well, particularly in C57BL/6J mice, a strain that is frequently employed in diabetes research [11]. Depending on the stage of reproductive life, the sex difference in diabetes prevalence is reversed [9]; that is, more men have the disease before puberty, while more women have it after menopause and in later life. The mechanisms underlying islet apoptosis induced by hyperglycemia remain unclear despite extensive research. Studies have shown that mitogen-activated protein kinases (MAPKs) regulate pancreatic islet function [12,13]. Investigations are still going on to determine the regulatory mechanisms of MAPKs in pancreatic islet function.

There are three main subgroups of mammalian MAPKs: p38 MAPKs, c-Jun NH_2_-terminal kinases (JNKs), and extracellular signal-regulated kinases 1 and 2 (ERK1/2) [14,15]. Studies have shown that MAPKs regulate pancreatic β-cell function. Youl et al. showed that ERK phosphorylation promotes quercetin’s role in protecting the INS-1β-cell line from oxidative damage [16]. On a chow diet, mice lacking p38δ exhibit enhanced insulin secretion, improved glucose tolerance, and activation of protein kinase D (PKD), a key regulator of insulin exocytosis [17]. The efficacy of metformin treatment in the STZ-induced T2D Sprague–Dawley rat model is linked to the downregulation of p38 MAPK and JNK phosphorylation [18]. These findings show that pancreatic β-cell function is negatively regulated by the JNK and p38 signaling pathways, while ERK phosphorylation positively regulates it. However, understanding the regulation of MAPK pathways in diabetes models would offer a significant understanding of the mechanisms underlying β-cell dysfunction.

Mitogen-activated protein kinase phosphatases (MKPs) dephosphorylate the MAPKs, rendering them inactive [19]. The MKPs comprise a family of 10 catalytically active enzymes that dephosphorylate MAPKs [19,20]. Although members of the MKP family have been shown to play diverse roles in metabolism [21,22,23], there is a paucity of information on the role of MKPs in pancreatic β-cell function and the development of T1D and T2D. One study found reduced MKP-5 expression in the pancreas and islet cells of mice fed a high-fat diet (HFD) compared to chow-fed mice [24]. While palmitic acid-induced apoptotic proteins like caspase-3, caspase-9, and PARP-1 are reduced in Rin-m5F cells when MKP-5 is overexpressed, insulin secretion is increased [24]. Another study found that parathyroid hormone-related protein (PTHrP) increases insulin secretion in mouse islet cells and MIN6 cells via activation of MKP-1 [16]. These findings suggest that MKPs are important regulators of pancreatic β-cell function. However, further research is needed to establish the role of MKPs in pancreatic β-cell dysfunction and the development of diabetes. Recently, we found that MKP-2 plays a major role in the development of obesity, insulin resistance, and nonalcoholic fatty liver disease [25]. However, the physiological role of MKP-2 in β-cell function and/or mass and development of diabetes remains largely unknown. The primary aim of this study was to determine the role of MKP-2 in the regulation of whole-body glucose homeostasis, while the secondary aim was the subsequent impact of this function on pancreatic islet function.

## 2. Materials and Methods

### 2.1. Reagents, Antibodies, and Immunoblotting

All reagents were purchased from standard chemical vendors. The following antibodies were used: phospho-JNK1/2 (#4668), phospho-ERK1/2 (#9101), phospho-Akt (S473) (#9217), phospho-Akt (T308) (#9275), ERK1/2 (4696), and α-tubulin (#2125) were obtained from Cell Signaling Technology (MA, USA). JNK-2 (#sc-7345) was obtained from Santa Cruz Biotechnology. Pancreas tissues from MKP-2 wild-type and MKP-2 knockout mice were isolated, processed, and immunoblotted as described [3].

### 2.2. Animal Studies

The University of Alabama Institutional Animal Care and Use Committee approved all animal studies (Protocol #2022.R04). The MKP-2 wild-type (WT) and MKP-2 knockout (KO) mice were kindly provided by Robin Plevin, University of Strathclyde, United Kingdom. Mice lacking MKP-2 have been genetically and metabolically characterized previously [26,27]. Male and female mice were housed under controlled temperature (23 °C) and lighting (12 h light, 12 h dark cycle, lights on at 0700 h) with free access to food and water. This study has been registered under animalstudyregistry.org. The in vivo animal experiments reported in this study are based on the ARRIVE guidelines [28]. The procedures, animals and resources used in this study are shown in Table 1 and Table 2.

#### 2.2.1. Animal Study Design 1a

To investigate the role of MKP-2 in the development of hyperglycemia and T1D, MKP-2 WT and KO male and female chow-fed mice ages 8 to 12 weeks were fasted for 4 h before receiving intraperitoneal (IP) injections of a low dose of streptozotocin (STZ) in a pH 4.5 citrate buffer (50 mg/kg per mouse body weight) for five days in a row. Every week, blood glucose levels and body weight were assessed. Mice that had blood glucose levels higher than 200 mg/dL five weeks after receiving an injection of STZ were classified as hyperglycemic [29,30]. The blood glucose levels were measured by a glucometer (Care Touch Blood Glucose Monitoring System).

#### 2.2.2. Animal Study Design 1b

To investigate the role of MKP-2 in the development of hyperglycemia and T2D, six-week-old MKP-2 WT and KO mice were fed a high-fat diet (HFD) for two weeks. The mice were placed on a low-dose intraperitoneal (IP) injection of 50 mg/kg of streptozotocin (STZ) in 0.1 M citrate buffer, pH 4.5, for five days in a row after they had fasted for four hours during the third week of the HFD. Every week, blood glucose levels and body weight were assessed. Mice that had blood glucose levels higher than 200 mg/dL five weeks after receiving an injection of STZ were classified as hyperglycemic [24,29,30]. The blood glucose levels were measured by a glucometer (Care Touch Blood Glucose Monitoring System). The control group (WT and KO) received an equal volume of citrate buffer.

#### 2.2.3. Experimental Unit 1c

Whole pancreatic tissues were isolated from STZ-treated and control T1D and T2D female and male MKP-2 WT and KO mice (n = 11–12 mice/group/sex; total n = 23). For each experiment, only one pancreas from each mouse (from either sex) was used in order to maintain statistical independence. Every single pancreas was regarded as an experimental unit [31].

#### 2.2.4. Sample Size 2

Because the mice used to donate experimental units for this study were inbred, subject variation need not be taken into account in the analysis. We can assume that the majority of the critical variables will be normally distributed, that we are primarily interested in changes of two-fold or more, and that the measures usually have an SD of no more than one-third of the mean in order to estimate the number of samples that will be required for each experiment. Years of experience with mouse in vivo metabolic studies lend credence to these hypotheses [32,33]. In light of these presumptions, we used *p* < 0.05 to reject the null hypothesis and aim for a power of 0.8 for each of the numerous independent variables we measured. According to this analysis, a two-tailed test requires a minimum of nine samples per group. These numbers were applied to mice of both genders.

#### 2.2.5. Inclusion and Exclusion Criteria 3

Experimental animals from both MKP-2 WT and MKP-2 KO from both sexes aged between six and twelve weeks were included in this study. Experimental units that did not fall under the age category were excluded from this study.

#### 2.2.6. Randomization 4

Randomization to assign animals to STZ-treated and control groups was not used in this study.

#### 2.2.7. Blinding 5

Four distinct researchers worked with each experimental animal in the following manner: A first investigator (NG) gave the treatment, as seen in Table 1. The assignment to the treatment group was only known to this investigator. Unaware of the treatment, the second investigators (MW and CS) and the first researcher separated experimental units from the animals. Experimental units were histologically analyzed by a third researcher (VU), who was not aware of the treatment. The histological pictures were examined by a fourth researcher (AF), who was not aware of the treatment.

**Table 1:** Mice in each group were given either citrate buffer (control) or streptozotocin (treated). Both T1D and T2D mice did not die during or after injection. Additionally, mice showed no symptoms of lethargy or hypoglycemia after receiving a STZ injection. Since there was no indication of hypoglycemia following the STZ injection, we did not use a 10% sucrose solution [34].

	Treatment	Genotype	Gender	N
T2D Treated	Streptozotocin	MKP-2 WT	F	12
	Streptozotocin	MKP-2 KO	F	12
	Streptozotocin	MKP-2 WT	M	10
	Streptozotocin	MKP-2 KO	M	10
T2D Control	Citrate buffer	MKP-2 WT	F	11
	Citrate buffer	MKP-2 KO	F	11
	Citrate buffer	MKP-2 WT	M	10
	Citrate buffer	MKP-2 KO	M	10
T1D Treated	Streptozotocin	MKP-2 WT	F	9
	Streptozotocin	MKP-2 KO	F	8
	Streptozotocin	MKP-2 WT	M	13
	Streptozotocin	MKP-2 KO	M	12
T1D Control	Citrate buffer	MKP-2 WT	F	9
	Citrate buffer	MKP-2 KO	F	7
	Citrate buffer	MKP-2 WT	M	11
	Citrate buffer	MKP-2 KO	M	11

#### 2.2.8. Outcome Measures 6

The following variables were measured in this study: body weight, tissue weight, blood glucose concentration, pancreatic islet size, protein phosphorylation, gene expression.

#### 2.2.9. Statistical Methods 7

All data are presented as mean ± SEM. Differences between groups were compared using Student’s unpaired two-tailed *t* test or one- and two-way analysis of variance (ANOVA). A post hoc test was performed using Bonferroni’s multiple comparisons using Prism 9 statistical software (GraphPad Software, La Jolla, CA, USA). A value of *p* < 0.05 was considered statistically significant. Gender was not considered a factor in the statistical analysis [25].

#### 2.2.10. Experimental Animals 8a

A total of one hundred sixty-six C57BL/6J mice (seventy-nine females and eighty-seven males) were used in this study. Six- to twelve-week-old mice were used.

#### 2.2.11. Genotype 8b

Mice lacking the expression of MKP-2 in all tissues were generated using targeted homologous recombination by removing exons 2–4 in collaboration with Genoway, Lyon, France using standard procedures [26]. These mice have been genetically characterized [26].

#### 2.2.12. Experimental Procedures 9a

Experimental procedures, reagents, and resources used in STZ-induced diabetes experiment are shown in Table 2.

**Table 2:** Experimental procedure, reagents, and resources used in STZ-induced diabetes experiment.

Procedures	Resources
**Pharmacological procedure** Drug: Streptozotocin; Fisher Scientific (Hampton, NH, USA) (#AAJ6160103). Dose: 50 mg/kg STZ per mouse body weight [34]. Site of injection: Intraperitoneal. Frequency of the administration: 5 days. Vehicle solution: 0.1 M Sodium Citrate buffer, pH 4.5. Pre-injection procedure: Mice fasted for 4 h before injection. Evidence of successful injection: Mice were hyperglycemic (>200 mg/dL) after 4 weeks of injection. Monitoring post-injection: Mice were checked regularly for sudden hypoglycemia or lethargy. Lethal effect of STZ injection during this study: No death of mice occurred during the experiment.	**Mouse model** Global MKP-2 WT and MKP-2 KO [26]. Genotyping of mice was performed by PCR using standard procedures [32].
**Blood glucose measurement** Blood was collected from the tail, and a blood glucose meter (Care Touch Blood Glucose Monitoring System) was used to measure blood glucose level. Basal blood glucose was measured before the injection, and weekly blood glucose level was measured post-STZ injection for 4 weeks.	**Reagents** Primer used for real-time PCR (see Section 2.5) Antibodies used for the immunoblotting: phospho-JNK1/2 (#4668), CST; phospho-ERK1/2 (#9101), CST; phospho-Akt (S473) (#9217), CST; phospho-Akt (T308) (#9275), CST; ERK1/2 (4696), CST; α-tubulin (#2125), CST; JNK-2 (#sc-7345), SCB. RNA Isolation kit: VWR (#101414-852) cDNA Reagent Kit: Thermo Fisher (Waltham, MA, USA) (#4368814)
**Euthanasia** Method of euthanasia: Mice fasted overnight, and CO_2_ asphyxiation followed by cervical dislocation [35]. Timing of euthanasia: 5–10 min. Euthanasia: Mice were exposed to CO_2_ asphyxiation until respiratory arrest was observed for 60 s. Cervical dislocation: Finger was placed at the base of the skull. Tail was pulled backward; forward and downward pressure was applied to the base of the skull [35]. Tissue collected after euthanasia: Pancreas, liver spleen, skeletal muscle, WAT, BAT, brain, heart, lungs. Storage of the tissue: Tissue was cut into small pieces and snap-frozen in liquid nitrogen, transferred to a sterile Eppendorf tube, and stored at −81 °C until further experimental use [36].	**Equipment and Software** Equipment used: Applied Biosystems 7500 Fast RT-PCR; Eppendorf Centrifuge 5430 R; BIO RAD (Hercules, CA, USA), Mini PROTEAN Tetra Cell, Cat # 1658005EDU; BIO-RAD, Mini Trans-Blot Cell, Cat # 1703930; BIORAD Chemidoc Imaging system (#12003154); Blood glucose meter: Care Touch Blood Glucose Monitoring System. Software used: Prism 9 statistical software (GraphPad Software, La Jolla, CA, USA).

#### 2.2.13. Experimental Procedures 9b

Body weight was measured before the STZ injection, and weekly body weight was measured post-STZ injection for four weeks. Basal blood glucose was measured before the STZ injection, and weekly blood glucose was measured post-STZ injection for four weeks.

### 2.3. Immunoblotting

RIPA buffer (25 mM Tris-HCl at pH 7.4, 150 mM NaCl, 5 mM EDTA, 1% NP-40, 0.1% SDS, and 1% sodium deoxycholic acid) supplemented with protease and phosphatase inhibitors was used to homogenize the pancreas and adipose tissues from MKP-2 WT and KO mice. On a shaker, the homogenates were lysed for 30 min at 40 °C. Subsequently, the lysates were separated at 20,800 g for half an hour at 40 °C. A Pierce bicinchoninic acid (BCA) protein assay kit (Pierce, Rockford, IL, USA) was used to measure the concentrations of proteins. Following SDS-PAGE resolution, lysates were transferred to a nitrocellulose membrane, incubated with phospho-specific antibodies, and then detected for fluorescence or enhanced chemiluminescence using a ChemiDoc MP imaging system (BioRad, CA, USA).

### 2.4. RNA Extraction and Real-Time PCR Analysis

Tissues derived from MKP-2 WT and MKP-2 KO mice were used to isolate total RNA using the E.Z.N.A HP kit (Omega Bio-Tek Inc., Norcross, GA, USA) according to the manufacturer’s instructions and as described [3]. Real-time quantitative PCR was performed as described [3], with the Applied Biosystems 7500 Fast RT-PCR system and SYBR Green gene expression master mix with the following primer pairs: *Pdx-1*, 5′-CCCCAGTTTACAAGCTCGCT-3′ and 3′-CTCGGTTCCATTCGGGAAAGG-5′;18S, 5′-ACCGCAGCTAGGAATAATGGA-3′ and 3′-GCCTCAGTTCCGAAAACCA-5′; *MafA*, 5′-AGGAGGAGGTCATCCGACTG and 3′-CTTCTCGCTCTCCAGAATGTG-5′; Bcl2, 5′-GTCGCTACCGTCGTGACTTC-3′ and 3′-CAGACATGCACCTACCCAGC-5′; FKN, 5′-ATT GGA AGA CCT TGC TTT GG-3′ and 3′-GCC TCG GAA GTT GAG AGA GA-5′. IL-1β, 5′-GACGGACCCCAAAAGATGAAGG-3′ and 3′-GAGGTGCTGATGTACCAGTTGG-5′; TNFα, 5′-CCCTCACACTCAGATCATCTTCT-3′ and 3′-GCTACGACGTGGGCTACAG-5′; Ins1, 5’-CGTGGCTTCTTCTACACACCCA and 3’-TGCAGCACTGATCCACAATGCC All relative gene expression levels were analyzed using the ΔCt method and normalized to 18S.

### 2.5. Histological Analysis of Tissue Sections

Tissues were isolated from STZ-treated and control T1D and T2D female and male MKP-2 WT and KO mice and then fixed in 4% paraformaldehyde in PBS and processed for paraffin sections and stained with hematoxylin and eosin as described [3].

## 3. Results

### 3.1. Female MKP-2 KO Are Hyperglycemic and Susceptible to STZ-Induced Diabetes

**Figure 1:**
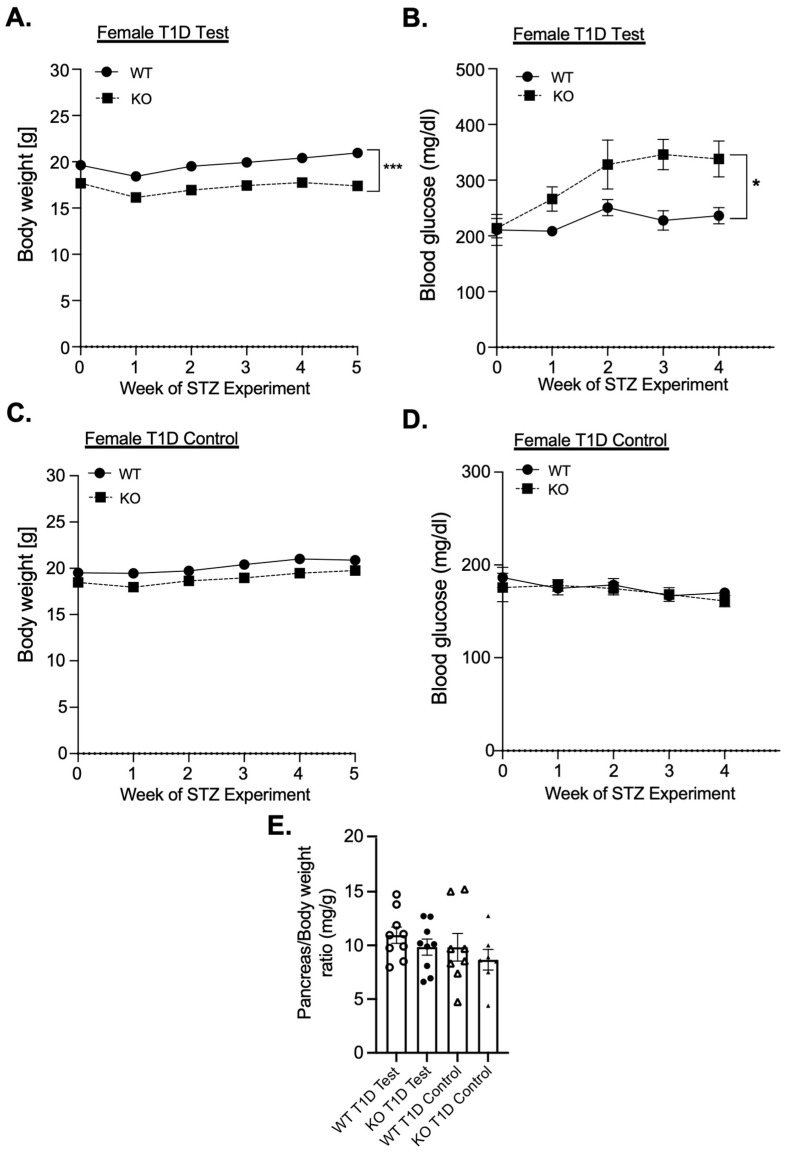
Body weight and blood glucose levels in female MKP-2-deficient mice in STZ T1D test. Weight curves of STZ T1D test (**A**) and control (**C**), blood glucose of STZ T1D test (**B**) and control (**D**), and pancreas weight (**E**) of MKP-2 WT and KO mice (n = 8–9 mice/genotype). Results represent the mean ± SEM; *: *p* < 0.05, ***: *p* < 0.0001 as determined by analysis of variance (ANOVA) with Bonferroni’s post-test for multiple comparisons.

In an examination of the role of MKP-2 in diabetes development in multiple-low-dose streptozotocin (STZ) mouse models using male and female chow-fed MKP-2 KO and MKP-2 WT mice aged 8–12 weeks, the body weight of female MKP-2 KO was significantly reduced during the course of STZ injections (STZ-treated) (Figure 1A). The body weight of STZ-treated male MKP-2 KO mice was significantly reduced compared to MKP-2 WT (Supplementary Figure S1A). Female MKP-2 WT and KO control mice exhibit comparable body weight in (Figure 1C); however, male MKP-2 KO control mice exhibit significantly reduced body weight compared with MKP-2 WT mice (Supplementary Figure S1C). Interestingly, STZ-treated female MKP-2 KO mice were significantly more hyperglycemic than female MKP-2 WT mice (Figure 1B); however, both male MKP-2 WT and KO mice exhibit hyperglycemia (>200 mg/dL) (Supplementary Figure S1B). This finding is exciting as most of the STZ-induced diabetes studies are performed on male mice, as studies have shown that female mice are resistant to STZ-induced diabetes [37], indicating that female MKP-2 KO mice are now more responsive to STZ-induced diabetes. Both female and male MKP-2 KO and WT control mice exhibit normal blood glucose levels (Figure 1D; Supplementary Figure S1D). These results suggest that MKP-2 deficiency promotes hyperglycemia in female mice in the T1D model.

**Figure 2:**
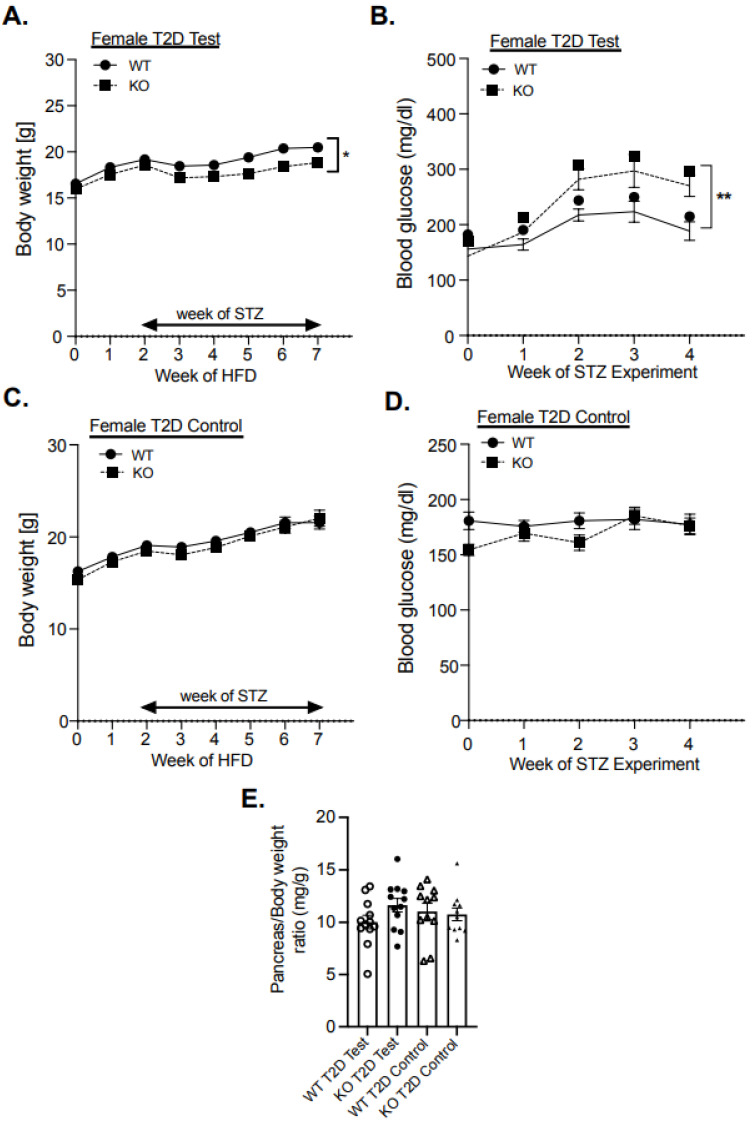
Body weight and blood glucose levels in female MKP-2-deficient mice in STZ T2D test. Weight curves of STZ T2D test (**A**) and control (**C**), blood glucose of STZ T2D test (**B**) and control (**D**), and pancreas weight (**E**) of MKP-2 WT and KO mice (n = 11–12 mice/genotype). Results represent the mean ± SEM; *: *p* < 0.05, **: *p* < 0.01, as determined by analysis of variance (ANOVA) with Bonferroni’s post-test for multiple comparisons.

To further investigate the role of MKP-2 in diabetes development, we utilized a T2D model. Six-week-old male and female MKP-2 WT and KO mice were fed a high-fat diet (HFD) followed by multiple-low-dose STZ. In T2D, STZ-treated male and female MKP-2 KO displayed reduced body weight compared with WT mice (Figure 2A); Supplementary Figure S2A). Female MKP-2 WT and KO control mice exhibit comparable body weight (Figure 2C); however, male MKP-2 KO mice exhibit significantly reduced body weight compared with MKP-2 WT mice in both STZ-treated and control mice (Supplementary Figure S2C). It has been previously reported that STZ injection causes a reduction in body weight in the T2D rat model [38]. Similarly, a recent study reported decreased body weight in STZ-treated MKP-5 KO mice in the T1D mouse model [39]. Interestingly, STZ-treated female MKP-2 KO mice were significantly more hyperglycemic than female MKP-2 WT mice (Figure 2B). Both STZ-treated male MKP-2 WT and KO mice were hyperglycemic (>200 mg/dL) (Supplementary Figure S2B), while control mice in both sexes and genotypes have normal blood glucose levels (Figure 2D); Supplementary Figure S2D). These findings imply that in the T2D model, MKP-2 deficiency induced hyperglycemia in female mice, suggesting that MKP-2 contributes to the maintenance of glucose homeostasis.

### 3.2. Impact of MKP-2 Deficiency in Islets of Female MKP-2 KO Mice in Diabetes

**Figure 3:**
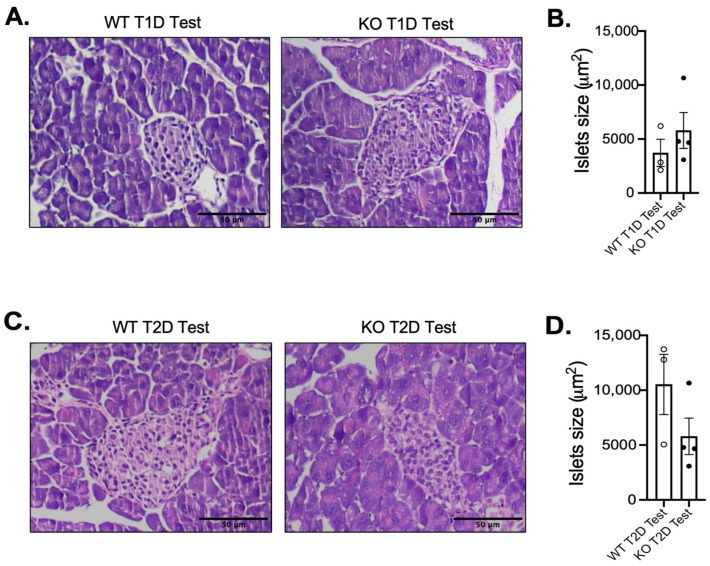
Islet morphology in female MKP-2-deficient mice in STZ T2D test. Representative H&E images of islet sections from T1D (**A**,**B**) and T2D (**C**,**D**) MKP-2 WT and KO female mice (n = 3–4) Islet size was analyzed using Image J software (version 1.54). Results represent the mean ± SEM.

Given that the development of diabetes is largely dependent on the maintenance of pancreatic islet function, we performed hematoxylin and eosin (H&E) staining of pancreatic islets from MKP-2 WT and KO T1D and T2D models. In the T1D model, H&E analysis of pancreatic islets demonstrated an increase in islet size in STZ-treated female MKP-2 KO mice that is not statistically bigger than that of the MKP-2 WT mice (Figure 3A,B). However, in the T2D model, the size of the pancreatic islets revealed by H&E analysis tended to be smaller in the STZ-treated female MKP-2 KO mice compared to the MKP-2 WT mice (Figure 3C,D). In both T1D and T2D, both sexes and genotypes exhibit comparable pancreas weight in the STZ-treated and control mice (Figure 1E and Figure 2E). The decrease in islet size may constitute STZ-damaged cells that lost their ability to secrete insulin in response to glucose, suggesting that MKP-2 activation in the islet cells can preserve them from cell death caused by STZ. These results suggest that MKP-2 activation may help improve islet size in T2D in females.

### 3.3. Enhanced MAPK Activity and Suppression of Pancreatic Development Gene Expression in Female MKP-2 KO Mice in T2D

**Figure 4:**
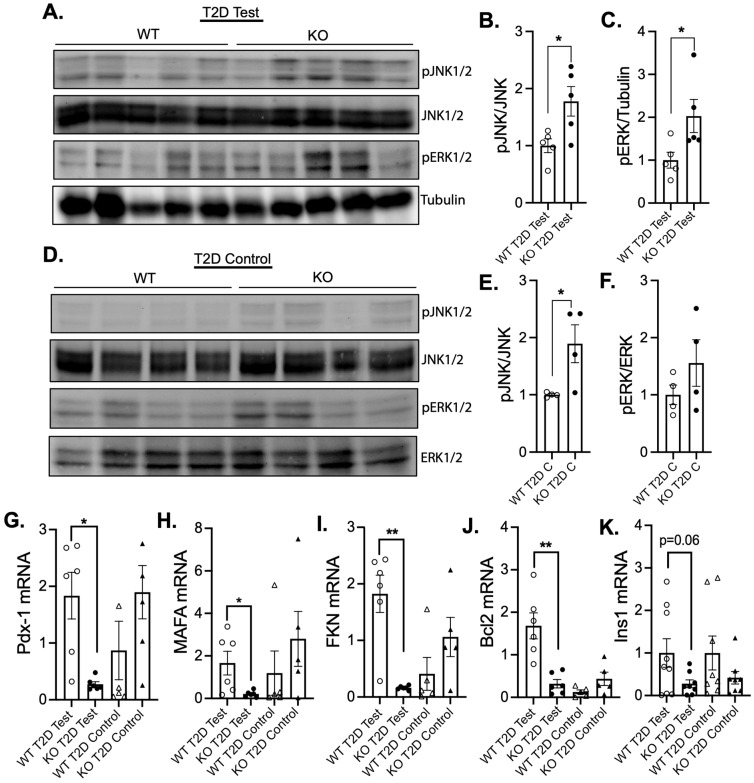
MAPK phosphorylation and pancreas gene expression in female MKP-2-deficient mice in STZ T2D test. Pancreas lysates from MKP-2 WT and MKP-2 KO mice were analyzed by immunoblotting with the indicated antibodies. Immunoblots were quantitated by densitometry for the levels of phospho-JNK1/2/JNK1/2 and phospho-ERK1/2/ERK1/2 (**A**–**C**) in the STZ T2D test and phospho-JNK1/2/JNK1/2 and phospho-ERK1/2/ERK1/2 in the STZ T2D control (**D**–**F**). Results represent n = 5/genotype. mRNA expression of Pdx-1 (**G**), MAFA (**H**), FKN (**I**), Bcl2 (**J**), and Ins1 (**K**) from STZ T2D (n = 6–9). *: *p* < 0.05, **: *p* < 0.01, test and control mice. Data shown are the mean ± SEM, as determined by analysis of variance (ANOVA) with Bonferroni’s post-test for multiple comparisons.

To substantiate the role of MKP-2 in the development of diabetes, we investigated the downstream targets that MKP-2 regulates in this process. Studies have shown that the MAPK pathway plays an important role in pancreatic islet function and the pathogenesis of islet apoptosis induced by hyperglycemia [12,13]. Since the only observed phenotype in T1D was hyperglycemia, we focused our analysis of the downstream targets on T2D. We measured the phosphorylation status of JNK and ERK in the pancreas of STZ-treated and control female MKP-2 KO and WT mice in T2D. In the STZ-treated mice, female MKP-2 KO mice displayed increased levels of JNK and ERK phosphorylation compared with WT mice (Figure 4A–C). However, in control mice, female MKP-2 KO mice displayed increased levels of JNK but not ERK (Figure 4D–F). Therefore, the lack of MKP-2 in the pancreas results in the upregulation of both JNK and ERK phosphorylation.

Activation of the MAPK pathway represents an important and fundamental mechanism through which islet function is achieved [40,41]. Therefore, we evaluated the levels of genes known to be important for pancreatic islet development and function, pancreatic and duodenal homeobox 1 (Pdx-1) and MAF BZIP transcription factor (MafA). We found that the expression of both genes was significantly reduced in the STZ-treated female MKP-2 KO pancreas compared with that in MKP-2 WT in T2D (Figure 4G,H). However, control female MKP-2 WT and KO mice exhibit comparable expression of Pdx-1 and MafA (Figure 4G,H). These results demonstrate that Pdx-1 and MafA are MKP-2 target genes, indicating a potential mechanism for reduced islet size in MKP-2 KO mice. Given that MaFA levels are more vulnerable to oxidative and metabolic stress than those of other islet-enriched transcription factors [42], it is possible that their decreased expression in MKP-2 KO mice under STZ conditions plays a role in the alterations in islet size and function linked to the development of hyperglycemia and T2D. Furthermore, we examined the expression of fractalkine (FKN), known to regulate pancreatic β-cell function. In the STZ-treated mice, we found that female MKP-2 KO mice exhibit significantly reduced FKN expression compared with wild-type mice (Figure 4I). Consistent with our findings, in vivo administration of FKN increased glucose tolerance [43]. No difference in FKN expression was observed in the control between MKP-2 WT and KO mice (Figure 4I). To examine whether hyperglycemia-induced cell death contributed to reduced islet size in female MKP-2 KO mice, we assessed the expression of the anti-apoptotic gene, Bcl-2, in the pancreas from STZ-treated female MKP-2 KO and WT mice. We found that the expression of Bcl-2 was significantly decreased in female MKP-2 KO mice compared with wild-type controls (Figure 4J). However, Bcl-2 expression was unchanged in the pancreas between female MKP-2 WT and KO control mice (Figure 4J). Male MKP-2 WT and KO mice in the T2D test exhibit comparable levels of Pdx-1, MafA, and Bcl2 (Supplementary Figure S3A–C). This suggests that MKP-2 KO female mice are susceptible to mitochondria-mediated apoptosis by attenuating anti-apoptotic factors leading to hyperglycemia and the development of T2D. According to a prior study, STZ activates JNK by inactivating phosphatases downstream of PARP-1 [44]. In MKP-2 KO mice, we observed increased JNK phosphorylation and decreased Bcl-2 expression, which is upstream of PARP-1. This suggests that MKP-2 mediates STZ-induced JNK activation. We then looked at the pancreatic expression level of *Ins1*, which is directly related to the amount of insulin generated. Consistent with hyperglycemia, we found Ins1 to be downregulated in the pancreas from MKP-2 KO mice (Figure 4K), suggesting a role for MKP-2 in the regulation of insulin secretion. These data indicate that in T2D, MKP-2 deficiency results in the downregulation of insulin-related gene expression in female mice.

### 3.4. Increased Adiposity and Insulin Resistance in Female MKP-2 KO Mice in T2D

**Figure 5:**
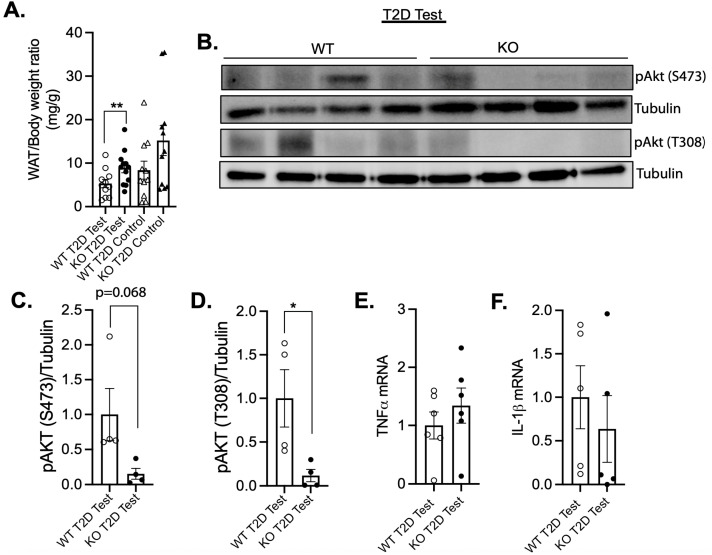
Increased adiposity and insulin resistance in female MKP-2-deficient mice in STZ T2D test. White adipose tissue weights of MKP-2 WT and MKP-2 KO in STZ T2D test and control mice (**A**) (n = 10–12/genotype). Adipose tissue lysates from MKP-2 WT and MKP-2 KO mice were analyzed by immunoblotting with the indicated antibodies. Immunoblots were quantitated by densitometry for the levels of phosphor-Akt/tubulin (**B**–**F**) in the STZ T2D test (n = 4/genotype). *: *p* < 0.05, **: *p* < 0.01, test and control mice. Data shown are the mean ± SEM, as determined by analysis of variance (ANOVA) with Bonferroni’s post-test for multiple comparisons.

We then considered whether impaired metabolic flux in peripheral tissues would be consistent with hyperglycemia and dysfunctional pancreas. Adipose tissue dysfunction promotes insulin resistance and impaired pancreatic islet dysfunction in T2D [45]. The finding that female MKP-2 KO mice show markedly enhanced adipose tissue mass under STZ-treated conditions compared with that for wild-type controls initially provided supporting evidence that the loss of MKP-2 could affect adipose tissue metabolism (Figure 5A). However, control female MKP-2 WT and KO mice exhibit comparable adipose tissue mass (Figure 5A). We next measured the phosphorylation status of Akt in the white adipose tissue (WAT) of STZ-treated female MKP-2 KO mice. The phosphorylation of Akt ^Ser473^ was also decreased but not statistically lower than that of wild-type mice (Figure 5B,C). We found that in the WAT of female MKP-2 KO mice, Akt ^Thr308^ phosphorylation was significantly reduced compared with MKP-2 WT mice (Figure 5B,D). These data demonstrate that in STZ-induced T2D, MKP-2 deletion results in reduced Akt activation that is associated with increased adiposity and insulin resistance in female MKP-2 KO mice. Along with cellular growth, survival, and apoptosis prevention, the Akt signaling pathway is a crucial regulator of islet cell function [46,47]. In the diabetic state produced by STZ injection, there was a decrease in Akt phosphorylation during rat placental development [48]. Diabetic muscle atrophy has also been linked to the inhibition of AKT signaling [49]. Consequently, our research indicates that inhibition of MKP-2 and downregulation of Akt signaling may be key mechanisms in STZ-induced hyperglycemia and diabetes. Next, we sought to determine the effects of inflammation on the development of insulin resistance in adipose tissue. The gene expression analysis of key inflammatory genes showed comparable levels of levels of TNFα and IL-1β in STZ-treated female MKP-2 KO mice and wild-type controls (Figure 5E,F).

## 4. Discussion

The purpose of this study was to investigate the physiological function of MKP-2 signaling in the pancreas and how it affects the development of hyperglycemia and diabetes. In patients with T2D, insulin injections have become the main clinical treatment and efficiently reduce hyperglycemia; however, these treatments frequently carry a risk of dysregulation of glucose metabolism [50]. Therefore, in order to find new therapeutic and preventive strategies, it is imperative to investigate the molecular mechanisms underlying the development of T2D. Understanding the pathogenesis of islet metabolic dysfunction resulting from hyperglycemia is crucial to our understanding of T2D development. Although the propagation of the MAPK pathway is critical for the promotion of signals that confer both islet life and death, the activation status and the different effects of various MAPK isoforms in the development of glucose tolerance and hyperglycemia have not been fully established [12,51]. Intriguingly, we found that female mice with a whole-body deletion of MKP-2 exhibit hyperglycemia in multiple-low-dose STZ mouse models. Consistent with the hyperglycemia, female MKP-2 KO mice exhibited reduced islet size. However, there was no significant difference in blood glucose levels between male MKP-2 KO and wild-type mice. This suggests that MKP-2 may have a sex-specific role in diabetes development. This is particularly interesting because sexual dimorphism in T2D is evident in many areas, such as pathophysiology, diagnosis, treatment, and prevention [52]. The sex differences in STZ sensitivity found in this study could be caused by additional factors. It is well known that STZ indirectly affects hormone levels and accessory sex organs [53]. STZ impairs testicular function and temporarily lowers serum testosterone levels in males [54,55]. Reduced viable oocytes and delayed oocyte maturation are the results of STZ-induced ovarian disruption in females [54,55]. Our current research provides a fresh perspective on the physiological significance of MKP-2 signaling, which is usually examined from a pathological perspective. It also shows that this pathway may have a sex-specific impact. The sex disparities described here might be important for designing MKP-2-signaling-pathway-focused human therapeutic trials in the future. Given that MKP-2 plays a role in the development of T2D, targeting MKP-2 specifically may offer significant therapeutic potential for the treatment of T2D, particularly in women. A thorough analysis of MKP-2 gene expression and splicing in human pancreatic tissues is necessary to uncover new avenues for the identification and assessment of therapeutic targets. It would be ideal to create a tissue-specific promoter system that targets T2D. Some cancer types are already being treated with this strategy [56]. It is necessary to further characterize the crucial roles that estrogens play in the processes that govern gender-dimorphic glucose regulation in MKP-2 KO mice. The levels of circulating hormones and their molecular regulation at the pancreatic tissue level should be included in the analysis of sex steroid levels in males and females in MKP-2 KO mice. Therefore, potential novel selective estrogen receptor modulators like MKP-2, which can mediate the protective effects of estrogens on body composition and glucose metabolism with negligible adverse effects on reproductive tissues, may be especially advantageous for menopausal women [57]. The discovery in this study raises the possibility that postmenopausal prevention of T2D may benefit from the activation of MKP-2 activity in islet cells.

Type 2 diabetes is characterized by dysregulation of major factors determining pancreatic islet survival and function [58,59]. Pro-survival pathways and genes essential for maintaining mature islet phenotype were downregulated in female MKP-2-deficient mice. The decreases in Pdx-1 and MafA, all crucial for preserving islet life and apoptosis, were linked to these abnormalities. Furthermore, we found that FKN, a regulator of β-cell insulin secretory function, was reduced in MKP-2 KO mice. Consistent with our findings, in vivo administration of FKN increased glucose tolerance [43]. Loss of MKP-2 resulted in reduced expression of the anti-apoptotic transcription factor Bcl-2. The consequences of decreased Bcl-2 in the pancreas of MKP-2-deficient mice are consistent with the observation that these mice exhibit reduced islet size in response to STZ injections. As a result, our research identifies MKP-2 as a regulator that might control the loss of islet survival and function.

Hyperglycemia induces reactive oxygen species (ROS) production in tissues, including the pancreas, which leads to oxidative stress [60,61]. Cellular oxidative stress induces JNK activation, which leads to DNA damage and cell death [62,63]. Furthermore, because there is insufficient expression of antioxidant enzymes, pancreatic islets are susceptible to oxidative stress [59]. Consistent with our study and others [64], we observed increased JNK phosphorylation and reduced Bcl-2 expression in the absence of MKP-2, suggesting hyperglycemia-induced JNK phosphorylation promotes islet cell death. Additionally, one study found that adding a dominant-negative form of JNK prevented oxidative stress-induced Pdx-1 translocation, which in turn prevented β-cell dysfunction in diabetes. Mice deficient in MKP-2 exhibit increased JNK activity that correlates with reduced Pdx-1 expression and islet size.

According to the current understanding of the pathophysiology of T2D, long-term excessive exposure to hyperglycemia and fatty acids causes adipose tissue dysfunction, which also contributes to pancreatic β-cell dysfunction by causing insulin resistance and lipid accumulation [65]. Here we found insulin resistance in MKP-2-deficient mice, which provides a mechanism for the increased adipose tissue mass. It is possible that MKP-2 contributes to pancreatic islet loss by acting indirectly on adipose tissue via lipolysis or a local adipokine/JNK pathway [66,67]. The mechanisms by which β-cell functional plasticity regulates insulin secretion in pathological conditions such as T2D are unclear; potentially examining the tissue-specific contribution of MKP-2 could identify novel pathways that regulate β-cell physiology to enhance insulin secretion. The sex hormones testosterone and estradiol may have contributed to the gender-biased phenotypes in female MKP-2 KO mice since they have an impact on islet cell function [68]. Research employing mice with ovariectomies should ascertain whether estrogen controls the gender-biased phenotype of MKP-2 KO.

### Limitations of This Study

According to our data, activating MKP-2 activity in the islets might offer a fresh approach to T2D prevention. This study does still have some limitations, though. This model has limitations because the deletion of MKP-2 in a whole-body setting raised the possibility of counter-regulatory effects from other tissues, making it impossible to draw firm conclusions from these studies. Therefore, examining the tissue-specific contribution of MKP-2 could identify novel pathways that regulate β-cell physiology to enhance insulin secretion. Also, the impact of MKP-2 deletion on the energy expenditure and physical activity of diabetic mice, which will assess MKP-2 KO mice susceptibility in a comprehensive manner, has not been studied.

## 5. Conclusions

In summary, we demonstrate the critical role of MKP-2 in the development of hyperglycemia and diabetes in vivo. Our results demonstrate that MKP-2 is an important regulator that might control the loss of islet survival and function. Additionally, these findings offer strong arguments for investigating gender-specific outcomes related to MKP-2 signaling regulating glucose metabolism and pancreatic islet function. Our research suggests that MKP-2 suppresses JNK and ERK activity, which leads to impaired pancreatic function and the development of diabetes. This finding raises the possibility that postmenopausal prevention of T2D may benefit from activation of MKP-2 activity in islet cells. To solve this problem, more research utilizing novel activators of MKP-2 is required. Furthermore, the cytoprotective properties of estrogens are one promising strategy to prevent islet apoptosis [69,70]. Estrogen’s feminizing effects restrict their clinical use in protecting islet survival in women and men. Provocatively, since estrogen uses non-specific extranuclear and membrane pathways, it may aid in the discovery and creation of novel regulators such as MKP-2/MAPK and ligands that safeguard islet cells and enable the retention of the positive effects of sex hormones in islets without having the mitogenic properties that lead to hormone-dependent disorders [69].

## Data Availability

The original contributions presented in this study are included in the article/Supplementary Material. Further inquiries can be directed to the corresponding author(s).

## Supplementary Materials

The following supporting information can be downloaded at: https://www.mdpi.com/article/10.3390/cells14040261/s1, Figure S1: Body weight and blood glucose levels in male MKP-2 Deficient Mice STZ T1D Test; Figure S2: Body weight and blood glucose levels in male MKP-2 Deficient Mice STZ T2D Test; Figure S3: Pancreas Gene Expression in Male MKP-2 Deficient Mice STZ T2D Test.
