# Supplementary material for: Mitogen-Activated Protein Kinase Phosphatase-2 Deletion Promotes Hyperglycemia and Susceptibility to Streptozotocin-Induced Diabetes in Female Mice In Vivo
Source: Cells. 2025 Feb 12;14(4):261. doi: 10.3390/cells14040261 (PMC11853640; doi:10.3390/cells14040261)
Supplement: Supplementary file 1 [file cells-14-00261-s001.zip › cells-3463009-supplementary.pdf]

Mitogen-Activated Protein Kinase Phosphatase-2 Deletion Promotes Hyperglycemia and Susceptibility to Streptozotocin-Induced Diabetes in Female Mice In Vivo

Nabin Ghimire[1], Morgan Welch[1,2], Cassandra Secunda[1,3], Alexis Fink[1], and Ahmed Lawan[1*]

[1]*Department of Biological Sciences, University of Alabama in Huntsville, Huntsville, Alabama 35899, USA*

#2 current address: Aviagen North America, Elkmont, AL 35620, USA

#3 current address: Alabama College of Osteopathic Medicine, Dothan, AL 36303, USA

Keywords: Hyperglycemia, diabetes, MKP-2, MAPK, islet

Running Title: Loss of MKP-2 promotes hyperglycemia

[*]To whom correspondence should be addressed.

Ahmed Lawan, Ph.D.,
University of Alabama in Huntsville,
Department of Biological Sciences
SST 369H, 301 Sparkman Drive,
Huntsville, AL 35899, United States,
Tel: 256-824-6264
Email: al0122@uah.edu

SUPPLEMENTARY FIGURE LEGENDS

SUPPLEMENTARY FIGURE 1: Body weight and blood glucose levels in male MKP-2 Deficient Mice STZ T1D Test. Weight curves of STZ T1D test **(A),** control **(C)** Blood glucose of STZ T1D test **(B)** control **(D),** Pancreas weight **(E)** of MKP-2 WT and KO mice (n=11-13 mice/genotype). Results represent the mean ± SEM; *; $p < 0.05$, *; $p < 0.01$, as determined by analysis of variance (ANOVA) with Bonferroni's post-test for multiple comparisons.

SUPPLEMENTARY FIGURE 2: Body weight and blood glucose levels in male MKP-2 Deficient Mice STZ T2D Test. Weight curves of STZ T2D test **(A),** control **(C)** Blood glucose of STZ T2D test **(B)** control **(D),** Pancreas weight **(E)** of MKP-2 WT and KO mice (n=9-10 mice/genotype). Results represent the mean ± SEM; *; $p < 0.05$, ***; $p < 0.0001$, as determined by analysis of variance (ANOVA) with Bonferroni's post-test for multiple comparisons.

SUPPLEMENTARY FIGURE 3: Pancreas Gene Expression in Male MKP-2 Deficient Mice STZ T2D Test

Pancreatic gene expression of Pdx-1 **(A),** Mafa **(B)** and Bcl2 **(C)** from male STZ T2D test MKP-2 WT and MKP-2 KO mice (n = 5 per genotype). Data shown are the mean ± SEM.

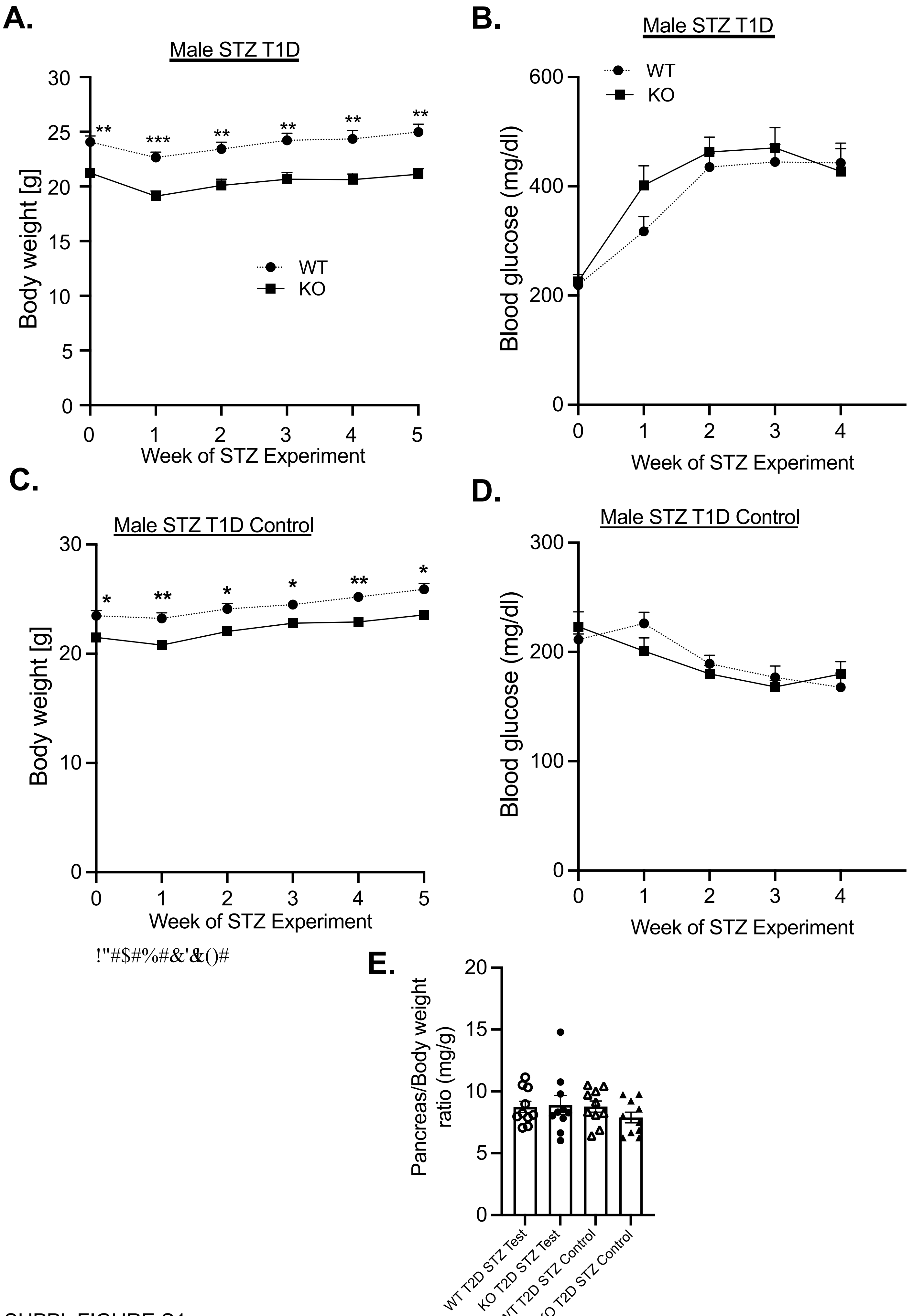

SUPPL FIGURE S1

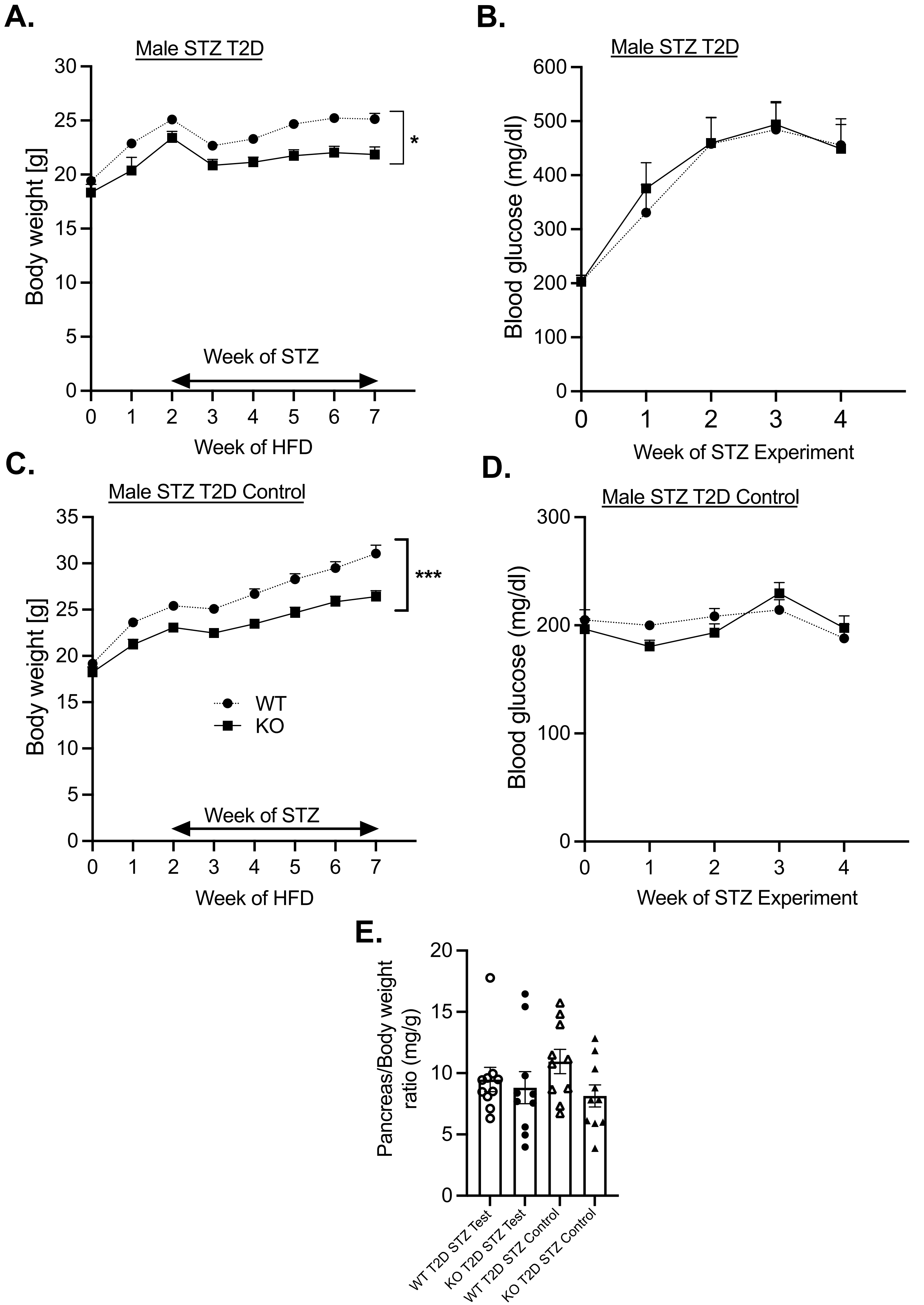

SUPPL FIGURE S2

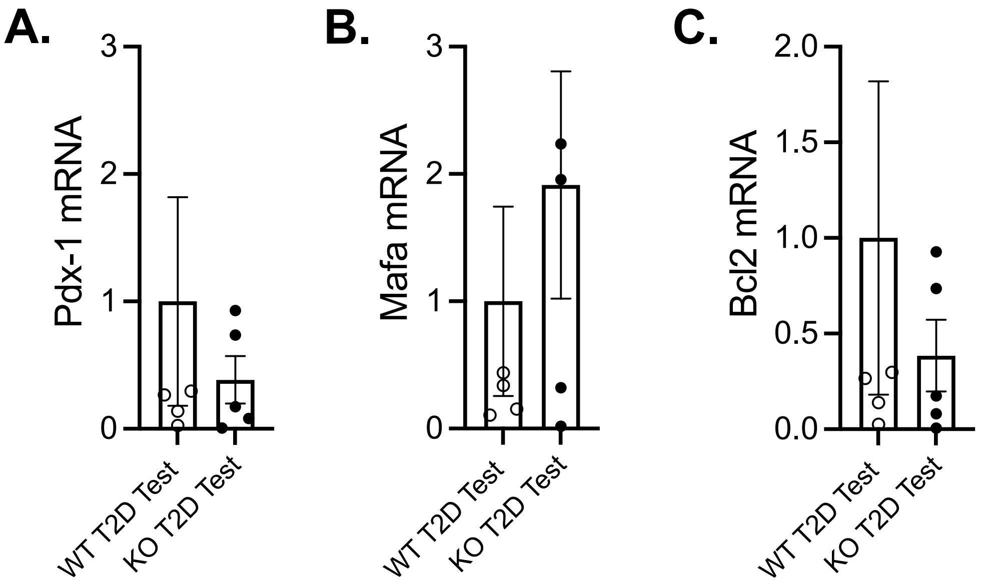

SUPPL FIGURE S3

May 4, 2022

TO: **Ahmed Lawan**

SUBJECT: Notice of Approval

Principal Investigator: **Ahmed Lawan**

Approval Date: **May 4, 2022**

UAH Approval Number and Proposal Codes:

Year.Code.ShortTitle

2022.R04.Lawan.Metabolism Referred to DMR (Designated Member Review) by Sharifa Love-Rutledge

The application described above was reviewed and by the UAH Animal Care and Use Committee, and referred for designated member review and has now been approved after suitable amendments. Congratulations!

This approval will be in effect for three years from the date of approval. If you have any significant amendments to make (change in PI, change in number or species, significant change in protocol, etc.) please let me know as soon as possible.

Best regards,

Roy Magnuson, Chair, UAH IACUC,
Associate Professor, Department of Biological Sciences,
University of Alabama in Huntsville
SC 369K, 301 Sparkman Drive, Huntsville, AL 35899
Email: Roy.Magnuson@uah.edu Cell: 256-724-0704
